# Catalytic Pyrolysis of Copper-Incorporated Nylon Fishing-Net Waste: Thermal Behavior, Evolved-Vapor Analysis, Kinetics, Thermodynamics, and Artificial Neural Networks

**DOI:** 10.3390/polym18172073

**Published:** 2026-08-26

**Authors:** Samy Yousef, Justas Eimontas, Nerijus Striūgas, Vilmantė Kudelytė, Deimantė Čepauskienė, Mohammed Ali Abdelnaby

**Affiliations:** 1 Department of Production Engineering, Faculty of Mechanical Engineering and Design, Kaunas University of Technology, LT-51424 Kaunas, Lithuania; 2 Laboratory of Combustion Processes, Lithuanian Energy Institute, Breslaujos 3, LT-44403 Kaunas, Lithuania; justas.eimontas@lei.lt (J.E.); nerijus.striugas@lei.lt (N.S.); vilmante.kudelyte@lei.lt (V.K.); 3 Laboratory of Heat–Equipment Research and Testing, Lithuanian Energy Institute, Breslaujos st. 3, LT-44403 Kaunas, Lithuania; deimante.cepauskiene@lei.lt; 4 Mechatronics Systems Engineering Department, October University for Modern Sciences and Arts-MSA, Giza 12451, Egypt; mohabdelnaby@msa.edu.eg

**Keywords:** copper-incorporated nylon fishing-net waste, catalytic pyrolysis, valuable products—caprolactam, 5-Cyano-1-pentene, artificial neural networks

## Abstract

In this research, the catalytic pyrolysis properties, kinetics, thermodynamic characteristics, and composition of the vapor evolved from the thermal decomposition of copper-incorporated nylon fishing net (CuFN) waste were investigated. The analysis was performed on CuFN composed mainly of nylon and copper (3 wt.%) as an anti-corrosion element. A comparative catalytic study was conducted using two types of zeolite catalysts, ZSM-5 (CuFNz) and Y-type (CuFNy). Reaction complexity in the presence of both catalysts was investigated through linear and nonlinear kinetic approaches, along with estimation of the relevant thermodynamic parameters. In addition, a well-trained artificial neural network was used to predict the catalytic thermal decomposition properties of both batches under untested heating conditions. Thermogravimetric results indicated that the catalyst type moderately influenced the decomposition profiles, with CuFNz achieving complete decomposition at 495 °C (44 wt.%), compared to 475 °C (52 wt.%) for CuFNy. Also, the type of catalyst did not affect the functional groups in TG-FTIR, which showed two main peaks at 1712 cm^−1^ (Carbonyl group) and 2933 cm^−1^ (C-H stretching band), but the alkyl C-H band was dominant in the case of CuFNy. Meanwhile, gas-chromatography–mass-spectrometry results indicated that caprolactam (88.21%) was a major GC compound in the CuFNz sample and 5-Cyano-1-pentene (70.43%) was dominant in the vapor of the CuFNy sample. However, the presence of the catalyst increases the complexity of the reaction, reflected by higher pyrolytic activation energies of 244.8 kJ/mol (CuFNz) and 296.3 kJ/mol (CuFNy). Moreover, the mysterious catalytic thermal decomposition of CuFN was fully recognized by the optimized ANN algorithm with R = 1. The study demonstrates that catalytic pyrolysis can convert CuFN into valuable products, including caprolactam using a ZSM-5 catalyst and 5-Cyano-1-pentene using a Y-type catalyst, potentially leading to significant environmental and economic benefits.

## 1. Introduction

Fishing nets are an essential component of marine environments, and their global market demand is estimated at approximately $2 billion [1]. Polyamide 6 (nylon) fishing nets are the most popular in the global net market, with an estimated market size of $1.5 billion in 2024 [2], representing more than 75% of global fishing net production. This high demand is a result of their being cheap, having a low weight, and exhibiting acceptable stability against chemicals, as these nets operate continuously in an environment rich in salts and strong acids [3,4,5]. However, they have very poor corrosion resistance, allowing pollutants and bacteria to settle and accumulate on their surfaces, causing biological contamination [6,7]. This accelerates their structural degradation in the form of tiny debris called microplastics [8,9], which have serious environmental repercussions and quickly end up as plastic waste [10,11]. To extend their service life, they are now reinforced with anti-corrosion elements in the form of a coating or filler [6,12,13,14]. Copper has shown great potential in this direction as it is added to polymer filaments during extrusion manufacturing, helping to reduce biological corrosion activity, keep mesh openings clean, and reduce cleaning and maintenance, nearly doubling their lifespan with good cost-effective performance [15,16,17,18,19]. Despite the promising results of these nets, the addition of copper makes their waste resistant to biodegradation, as well as to the decomposition of the plastic itself, which is usually treated as municipal solid waste and ends up in landfills [20]. Copper is also classified as a heavy metal that can leach into the soil and from there into groundwater, contaminating it with heavy metals [21,22]. According to the current regulations for managing net waste, there is no specific system for managing it. It is typically classified as plastic waste and is commonly referred to as marine or ocean plastic waste [23,24], along with fishing net (FN) waste and a portion of marine plastic waste, which usually ends up in landfills or is incinerated, the most common traditional methods for disposing of plastic waste [25]. Since many countries have recently adopted legislation to halt such practices due to their economic inefficiencies and emissions, investments have been made in some advanced technologies to treat marine plastic waste in general and FNs individually [11,26]. These solutions suggested using FNs for textile reproduction [27]. Others have suggested using fibers derived from FNs in the production of porous asphalt mixtures for the purpose of building sustainable road paving [28]. They can also be utilized in the transition zone between concrete and the fibrous mortar composite [29]. It also has applications in enhancing the microstructure of mortars and the mechanical performance of gypsum composites and concrete and cementitious composites [30,31,32,33]. In another solution, waste fishing nets were used in the production of clean energy (hydrogen) [34]. Since caprolactam is the main compound of nets, many studies have focused on recovering it using chemicals and pyrolysis as an eco-friendly approach, which helps in closing the loop. Chemical recycling processes (alkaline and acidic hydrolysis and supercritical processes) have been suggested for this purpose and for the extraction of caprolactam from polyamide 6 in general and have shown good performance in terms of high economic performance [35]. However, it requires too many chemicals and requires energy-intensive separation. Therefore, thermal treatment using solutions of pyrolysis has received a lot of attention and has proven to be very effective in extracting caprolactam. In fact, research on this topic began very early in this century, starting with a study of the thermal behavior of fresh materials and FNs in a micro-scale tubular pyrolysis reactor, which showed that the principal pyrolysis region lies in the range of 340 and 440 °C [36]. Then, the thermal decomposition of FN was investigated using a thermogravimetric system (TGA), Fourier transform infrared (FTIR) spectroscopy, and a pyrolyzer (Py)–gas chromatography/mass spectrometry (GC/MS) system as analytical reactors under the effect of several factors such as seawater aging [37]. Also, pyrolysis of FN was examined using Py-GC/MS at various temperatures (400–700 °C), and the results indicated that 64–86% of alternative caprolactam could be produced from the process [38]. In addition, FN pyrolysis over different catalysts like scallop shells, carbon dioxide, and zeolite is used to improve caprolactam recovery (up to 83% and 60.31% from typical pyrolysis without catalysts) using an analytical reactor, and its decomposition mechanism is determined based on extensive kinetic studies of its behavior [39,40,41]. For upscaling, studies have started to focus on performing pyrolysis experiments using real and large-scale reactors for converting FNs over catalysts into value-added chemicals and energy products, especially caprolactam compounds, and determining their productivity and composition. When ZSM-5 and Y-Type catalysts were used, the conversion process successfully recovered 96% of caprolactam abundance at 700 °C (Y-Type) [42], while metallized seaweed-derived bio-char catalysts contributed to increasing the liquid pyrolysis product of nets to 78.36 wt.% [43]. Meanwhile, the FN catalytic pyrolysis process in a quartz-based pyrolysis reactor in an ambient CO_2_ reaction using a seashell-derived catalyst resulted in 77.7% caprolactam at 500 °C [44]. Copper-incorporated nylon fishing-net (CuFN) waste is a modern type of FN and represents an environmental challenge due to the persistence of nylon and the potential release of copper into marine environments [45]. Pyrolysis provides a promising route for CuFN valorization by converting the nylon fraction into caprolactam-rich vapor while retaining copper in the solid residue for subsequent recovery. Recent studies reported caprolactam abundances of up to 89.45% in the pyrolysis vapor of CuFN, with the copper fraction remaining largely in the solid residue and subsequently recoverable through a chemical leaching process [46,47]. To enhance the abundance of caprolactam in the released vapor up to 96%, chemical leaching was used to extract the copper fraction before exposure to pyrolysis as the main treatment [48], which confirms that the pyrolysis process has high potential in the treatment of CuFN. However, the catalytic pyrolysis of CuFN has not yet been investigated, which requires future research to discover its possibilities in this regard. This is reinforced by the fact that studies in this field have confirmed that the catalytic pyrolysis process of FN can increase caprolactam extraction and reduce the complexity of the reaction or perhaps develop new value-added chemicals (depending on the catalysts used) that need to be discovered [41,42,43,44]. To explore this, this research aims to discover the catalytic pyrolysis of CuFN and simulate its thermal behavior, kinetics, and thermodynamic analysis. The CuFN pyrolysis experiments were carried out using a thermogravimetric analyzer (TGA) in the presence of commercial ZSM-5 and Y-Type catalysts, which had previously been used for this purpose with FN [42,43]. The effect of catalyst composition on the released catalytic evolved vapors was noted using TG-FTIR and GC/MS spectroscopy. Based on the catalytic thermal decomposition measurements at varying heating rates, the kinetic–thermodynamic behavior of CuFN over the listed catalysts was simulated, followed by prediction of their TGA data under previously unmeasured heating conditions using a well-trained artificial neural network (ANN).

## 2. Experiment and Methodology

### 2.1. CuFN Feedstock Preparation

The CuFN feedstock tested in the present experiments was sourced from the Baltic region. This CuFN consists of nylon and copper (3 wt.%) and contains 87.4% volatile matter and 59% carbon, as shown in our previous work [46]. The supplied CuFN was initially sun-dried, cut into small pieces, and crushed into short segments. The crushed material was subsequently milled and sieved to obtain fine particles with a size of less than 200 µm. The milled samples were then dried under sunlight at 25 ± 2 °C and 86 ± 3% relative humidity for 72 h before the experiments. ZSM-5 and Y-Type catalysts were selected for the present work as they showed a high caprolactam yield [41,42]. Both catalysts were obtained from Sigma-12 Aldrich, and their properties have been reported elsewhere [49]. The crushed CuFNs were mixed with 20 wt.% of ZSM-5 (CuFNz) and Y-type zeolite (CuFNy) catalysts, where this ratio is considered the optimal mixing ratio to provide higher caprolactam production [41]. The thermal decomposition of CuFN samples was initially investigated, and the resulting vapors were analyzed using TG-FTIR and GC/MS techniques. Subsequently, the kinetics of the catalytic thermal decomposition of CuFN were modeled in terms of kinetics and thermodynamics, and finally, its decomposition profile was predicted using ANN machine learning.

### 2.2. Thermogravimetric Analysis

The thermal degradation of CuFNz and CuFNy samples was determined using TGA (NETZSCH). Approximately 10 mg of each CuFN sample was analyzed under a nitrogen atmosphere acting as a non-reactive medium with a pumping flow rate of 60 mL/min. The measurements were taken from room temperature to 900 °C at heating rates (*β*) ranging from 5 to 30 °C/min in increments of 5 °C/min. The effect of these conditions on the weight loss of CuFNz and CuFNy samples was recorded to plot their TGA curves, followed by plotting their derivative thermogravimetric (DTG) profiles using Proteus software. The TGA and DTG data measured subsequently were used to calculate the catalytic pyrolysis index (CPI) for both CuFN samples using formula (1) [50]. In this formula, *R*_max_, *R*_avg_, and *M*_f_ items are defined as the maximum thermal catalytic decomposition rate (%/°C), average mass loss rate of CuFN samples, and residual mass (wt.%) at the end of reaction, respectively. Meanwhile, *T_i_*, *T_m_*, and ${\Delta T}_{1/2}$ refer to initial devolatilization temperature (°C), the maximum decomposition peak (°C), and temperature range corresponding to R/R_peak_ = 0.5 [51].

(1) $$\mathrm{CPI}=\frac{\left(-R_{max}\right)\times \left(-R_{avg}\right)\times M_{f}}{T_{i}\times T_{m}\times {\Delta T}_{1/2}}$$

### 2.3. Analysis of the Evolved Vapor

The functional groups of the evolved-vapor phase that evolved throughout the catalytic pyrolysis of CuFNz and CuFNy samples in the key decomposition zone (detected via DTG profiles) under the applied heating conditions were examined via a TG-FTIR integrated system. To ensure leak-free transfer of evolved vapors and their compounds, TGA vapors were directly introduced into the FTIR instrument through an infrared gas cell with a potassium bromide inner window and a zinc selenide outer window. All TG-FTIR measurements were acquired using 32 scans per wavelength and a liquid nitrogen-cooled mercury and cadmium detector, while the evolved vapors from catalytic pyrolysis of CuFN samples were analyzed for their composition and quantity using a GC/MS system (Agilent 7890 A GC coupled with a 5975 MSD). The GC/MS measurement was performed in a high-purity helium atmosphere at ≥99.9999 % and a pressure of 20 psi. The evolved vapors were sent to the GC/MS examination system through the quartz tube inlet with the help of an external pump for injection purposes at 250 °C. The chromatographic separation process was performed using an HP-5MS column (inner diameter: 0.25 μm; outer diameter: 250 μm; length: 60 m) over a temperature range of 40 °C to 270 °C with a ramp of 10 °C/min. The mass spectra of the evolved vapors were recorded over a range of 30–600 *m*/*z*, and compound identification was implemented by comparison with the National Institute of Standards and Technology (NIST) database. Finally, to ensure the accuracy, reliability, and reproducibility of the experimental results, the TGA, TG-FTIR, and GC-MS instruments were calibrated, and their performance was verified in accordance with the manufacturer’s recommendations prior to analysis. To assess experimental repeatability, the CuFNz sample was analyzed in triplicate at a heating rate of 5 °C/min under identical experimental conditions. The repeated measurements showed a deviation of less than 1% for weight loss in TGA, transmittance in FTIR, and relative peak area in GC-MS, confirming excellent repeatability. Following this validation, experiments at the other selected heating rates were performed once under the same controlled operating conditions. This approach ensured consistent and reliable measurements while minimizing experimental variability.

### 2.4. Kinetic and Thermodynamics Analysis

The catalytic thermochemical decomposition process of CuFNz and CuFNy samples was revealed by the single reaction approach, as DTG analysis showed a single major peak even after the inclusion of copper (multi-component system: nylon and copper) in the reaction [41,46]. Model-free techniques are the most common approaches for this, and they are classified into linear methods and modeling processes, which do not require the assumption of a specific reaction mechanism, unlike model-fitting methods, which require several assumptions about the nature of the reaction mechanism and its orders [52]. Accordingly, isoconversional (model-free) techniques were applied in the current work to simulate the catalytic thermochemistry of CuFN using KAS (Kissinger–Akahira–Sunose), FWO (Flynn–Wall–Ozawa), and FM (Friedman), which allowed us to investigate the impact of adding ZSM-5 and Y-Type catalysts on the reaction complexity in the form of pyrolytic activation energy (Ea) and pre-exponential factor (A) items [53]. Using these approaches, Ea at a given conversion rate can be computed by fitting the distinctive relationship outlined for each model based on formulas (2)–(4). The slope of each fitted line then provides the Ea value according to the following expressions: KAS (-Ea/R), FWO (-1.0516Ea/R), and FM (-Ea/R) [54,55]. The complexity of the interaction was also determined using the Vyazovkin (VN) algorithm as a popular nonlinear strategy developed based on an optimization process to minimize the objective function in MATLAB software until constant Ea values are obtained, which increases the accuracy of the results with the help of Equation (5) [56]. This variety in the models used, as well as the working mechanisms, provides more space to select the most suitable model to simulate the kinetic catalytic pyrolysis of CuFN. Finally, using the estimated *E_a_* and *A* items obtained from the kinetic models, the thermodynamic parameters of CuFN catalytic pyrolysis—namely enthalpy (ΔH), Gibbs free energy (ΔG), and entropy (ΔS)—were calculated according to Equations (6)–(8) [57]. In these thermodynamic formulas, *K_B_* = 1.3819 ×10^−23^ J/K, *R* = 8.314 J/mol K, and *h* = 6.6269 ×10^34^ Js [58,59].

(2) $$\ln\left(\frac{\beta }{T^{2}}\right)\;=ln\left(\frac{AR}{E_{a}g\left(y\right)}\right)\;-\frac{E_{a}}{RT}$$

(3) $$ln\beta \;=ln\left(\frac{AE_{a}}{Rg(y)}\right)\;-5.331-1.0516\frac{E_{a}}{RT}$$

(4) $$ln[\beta \left(\frac{dy}{dT}\right)]=ln\left(Af\left(y\right)\right)-\left(\frac{E_{a}}{RT}\right)$$

(5) $$g\left(y\right)=\int_{0}^{y}\frac{dy}{f(y)}=A\int_{0}^{t}exp(-E_{a}/RT)dt$$

(6) $$\Delta H=E_{a}-{\mathrm{RT}}_{m}$$

(7) $$\Delta G=E_{a}+{\mathrm{RT}}_{m}\;\ln\left(\frac{K_{B}T_{m}}{hA}\right)$$

(8) $$\Delta S=\frac{\Delta H-\Delta G}{T_{m}}$$

### 2.5. Artificial Neural Network Analysis

The thermal decomposition behavior of any feedstock is significantly affected by many parameters, and heating rates are one of them [60]. As is well known, there are a huge number of heating rates, and to know the thermal decomposition of all of them, a huge effort of laboratory tests must be carried out, which consumes a large amount of time and cost. To overcome this problem, ANN was used as an advanced learning mechanism for training and prediction in the present research to predict the catalytic thermochemical behavior of CuFN under untested pyrolytic heating rates. This is because ANN has an exceptional capacity to model nonlinear relationships and to treat the noisy measured data that can be generated from TGA measurements through rigorous training and the use of advanced algorithms [61]. In the present research, an ANN model with a multi-layer architecture was built via MATLAB software and optimized using Levenberg–Marquardt back-propagation (LMB) configuration. The developed model was divided into three layers dedicated to an input layer, a processing layer (for capture of complex data), and an output layer. Temperature, heating rate, and conversion rate (0.1–0.9) served as the key input data, while the resulting TGA weight loss versus catalytic reaction temperature was the primary output [62]. The model was trained, tested, and validated at 70%, 15%, and 15%, respectively. These percentages are taken from the literature, which has demonstrated good prediction performance [63]. The developed ANN algorithm was trained and optimized based on mean absolute error (MAE), mean bias error (MBE), root mean square error (RMSE), and correlation coefficient R^2^, which can be expressed as shown in Equations (9)–(12) [64]. Finally, the optimized ANN algorithm predicted the catalytic thermal decomposition of CuFN at 17 °C/min, a pyrolytic heating rate that was not observed in laboratory experiments.

(9) $$\mathrm{MAE}=\frac{1}{N}\sum_{i=1}^{N}\left|H_{i}-H_{i,mol}\right|\;$$

(10) $$\mathrm{MBE}=\frac{1}{N}\sum_{i=1}^{N}\left(H_{i}-H_{i,mol}\right)$$

(11) $$\mathrm{RMSE}=\sqrt{\frac{1}{N}\sum_{i=1}^{N}{\left(H_{i}-H_{i,mol}\right)}^{2}}$$

(12) $$R^{2}=1-\sum_{i=1}^{N}{\left(H_{i}-H_{i,mol}\right)}^{2}/\sum_{i=1}^{N}{\left(H_{i}-H_{i,avg}\right)}^{2}$$

## 3. Results and Discussion

### 3.1. Thermogravimetric Analysis

**Figure 1:**
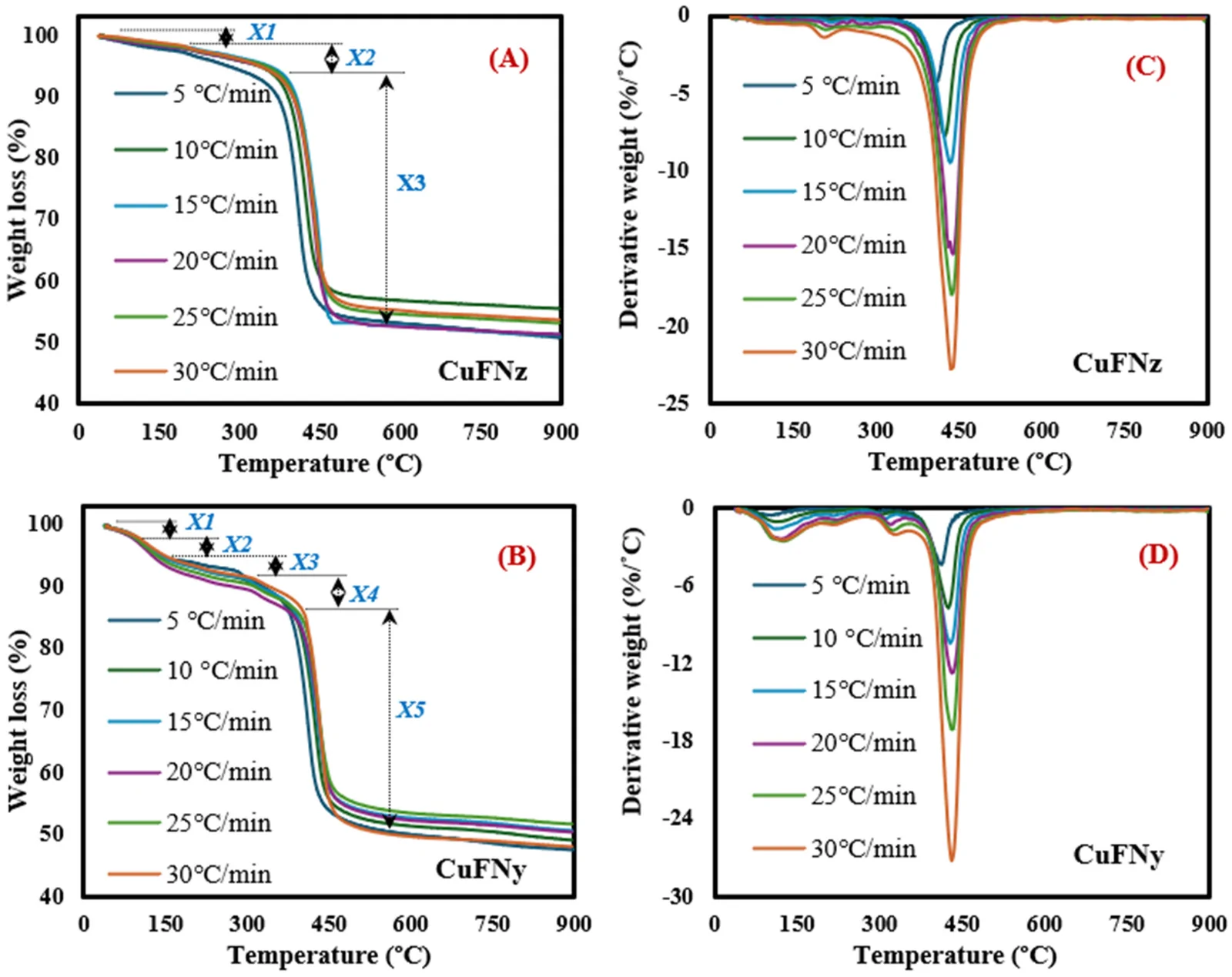
(**A**,**B**) TGA and (**C**,**D**) DTG relationships of CuFNz and CuFNy samples.

Figure 1 displays the measured TGA and DTG relationships of the CuFNz and CuFNy samples. According to the TGA analysis, the CuFNz sample (Figure 1A) undergoes complete decomposition up to 495 °C, with a total weight loss of approximately 44 wt.%. Also, it was noticed that the CuNz decomposed into three distinct regions described as X1 up to 200 °C, X2 up to 375 °C, and X3 up to 495 °C, with a mass loss estimated at 2 wt.%, 5 wt.%, and 37 wt.%, respectively. The first region refers to the release of moisture content in the feedstock and catalyst [41]. The second region refers to the release of light volatile content and copper debris [46], while the last stage, with significant mass drop, refers to active catalytic pyrolysis of this sample. On the other hand, the TGA of the CuFNy sample (Figure 1B) showed a slightly lower decomposition temperature (475 °C) and higher overall weight loss (52 wt.%). Also, the decomposition process of the CuFNy sample occurs in five distinct stages distributed as follows: X1 up to 120 °C (3 wt.%), X2 up to 170 °C (3 wt.%), X3 up to 320 °C (3 wt.%), X4 up to 390 °C (4 wt.%), and X5 up to 575 °C (35 wt.%). In this sample, the moisture content is released in the X1 and X2 regions, followed by the release of light volatile matter content in X3, then copper debris separation in X4. In the final stage (X5: active catalytic pyrolysis), the released vapor started to be emitted with high intensity and was accompanied by intensive reactions combined with significant mass loss. Once the active catalytic pyrolysis of both samples had ended, a solid carbonaceous formation stage (solid residue) was developed that had undercomposed fractions, including copper debris and char fraction [46]. Simultaneously, the DTG curves of both samples exhibited a solitary decomposition peak, and their characterizations under various heating rates are summarized in Table 1. As shown in the table, the maximum decomposition temperature (Tpeak) is estimated in the range of 409–441 °C (CuFNz) and 412–433 °C (CuFNy). In fact, this range is lower than the results obtained from typical pyrolysis without a catalyst, which was up to 456 °C [46]. It was observed that an increase in heating rate led to a progressive shift in the peak toward higher temperatures, due to thermal hysteresis delaying decomposition of CuFN samples [64]. Finally, CPI calculations showed that the CuFNz sample had an average CPI of 1.27 × 10^−4^ versus 1.46 × 10^−4^ for the CuFNy sample (Table 1), meaning that the catalytic thermal decomposition of CuFNy provides higher performance with a 15% improvement, which enhances the synthesis of more value-added chemicals [65].

### 3.2. TG-FTIR Analysis of the Evolved Vapor

**Figure 2:**
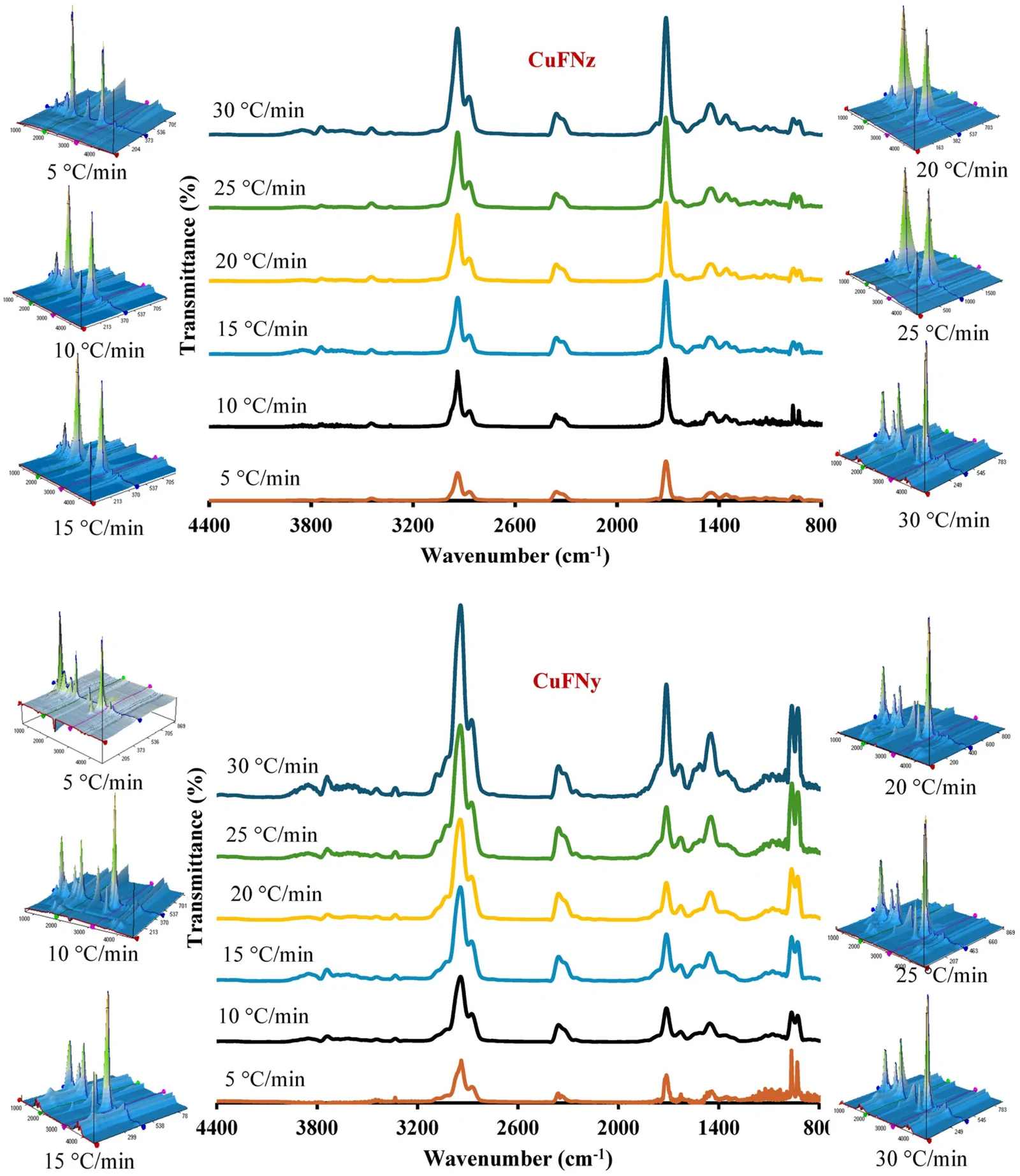
TG-FTIR spectra of the evolved vapors during the pyrolysis of CuFNz and CuFNy samples.

The functional groups of the evolved vapor from the principal catalytic pyrolysis region of CuFNz and CuFNy samples under various heating steps are presented in Figure 2. The analysis of the vapors of the CuFNz sample indicated that its vapors consisted of 1712 cm^−1^ (a C=O bond refers to a carbonyl group) and 2933 cm^−1^ (C-H stretching) as major functional groups. There were also some weak peaks, at 916–964 cm^−1^ (OH bending), 1436 cm^−1^ (C–H bond refers to aromatic), and 2308 cm^−1^ (the C=O stretching band refers to CO_2_), as shown in its two-dimensional (2D)-FTIR spectra. These FTIR spectra are largely identical to those obtained from the functional groups of FN and CuFN [41,46]. Meanwhile, the 2D-FTIR spectra of vapors of the CuFNy sample showed FTIR spectra almost like those obtained from the decomposition of the CuFNz sample, but the alkyl C-H stretch (2948 cm^−1^) peak became dominant. Also, the intensity of the C=O bond (carbonyl) (1712 cm^−1^) decreased significantly and became almost equal to the OH bending peak (923–968 cm^−1^). It was observed that the intensity of all functional groups of both samples became stronger and sharper with the gradually enhanced heating rate because of an improvement in the rate of heat flux generation, which promotes the degradation rate of CuFNz and CuFNy molecules and limits the unwanted reactions [46]. This feature also helps in providing a smooth spectrum, as shown in three-dimensional (3D)-FTIR. These results demonstrate that the catalytic pyrolytic process produces vapors rich in esters, alkyl compounds, and certain by-products, which can be identified using GC/MS analysis.

### 3.3. GC/MS Analysis of the Evolved Vapor

**Figure 3:**
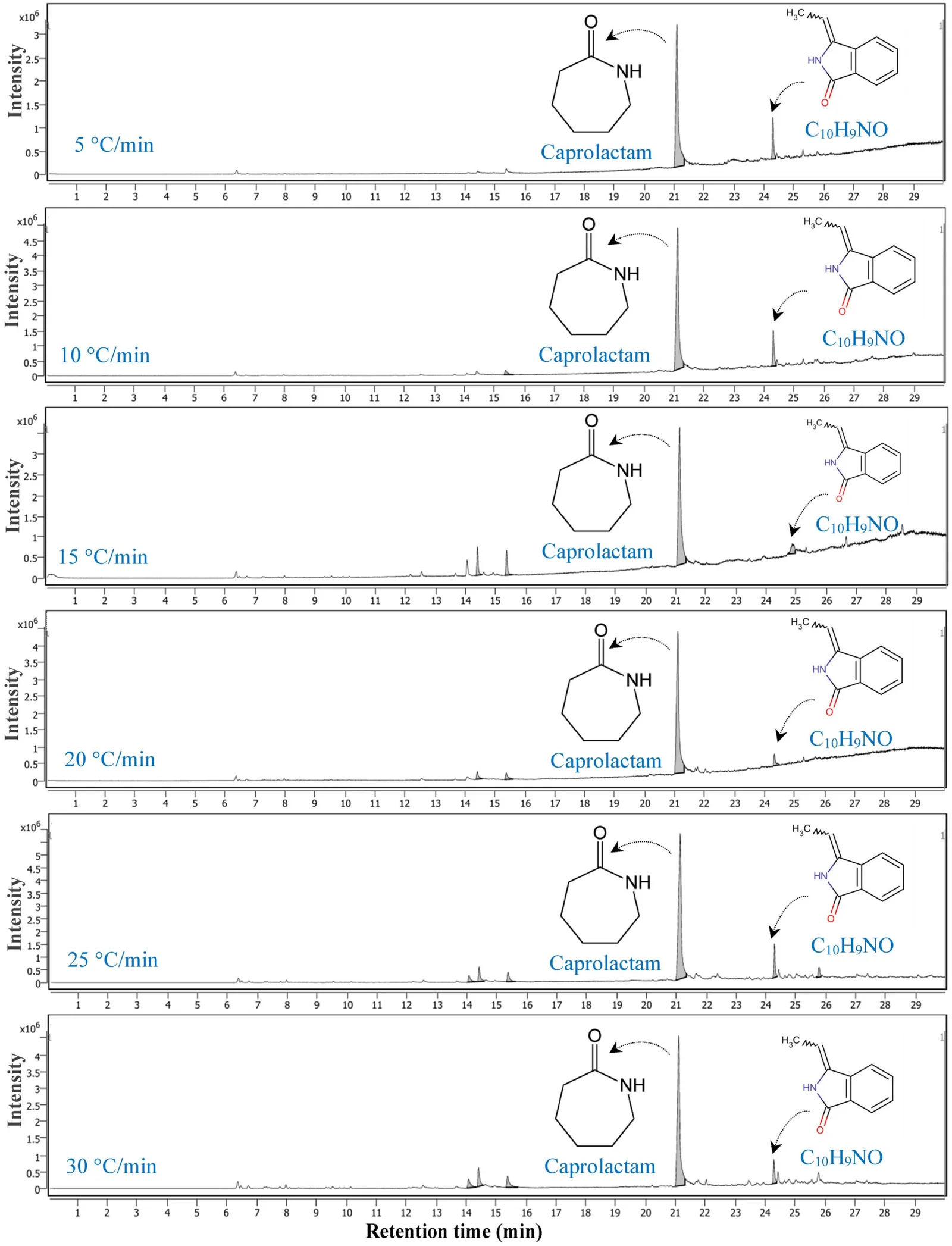
GC/MS chromatogram of the evolved vapors from the catalytic decomposition of the CuFNz sample.

**Figure 4:**
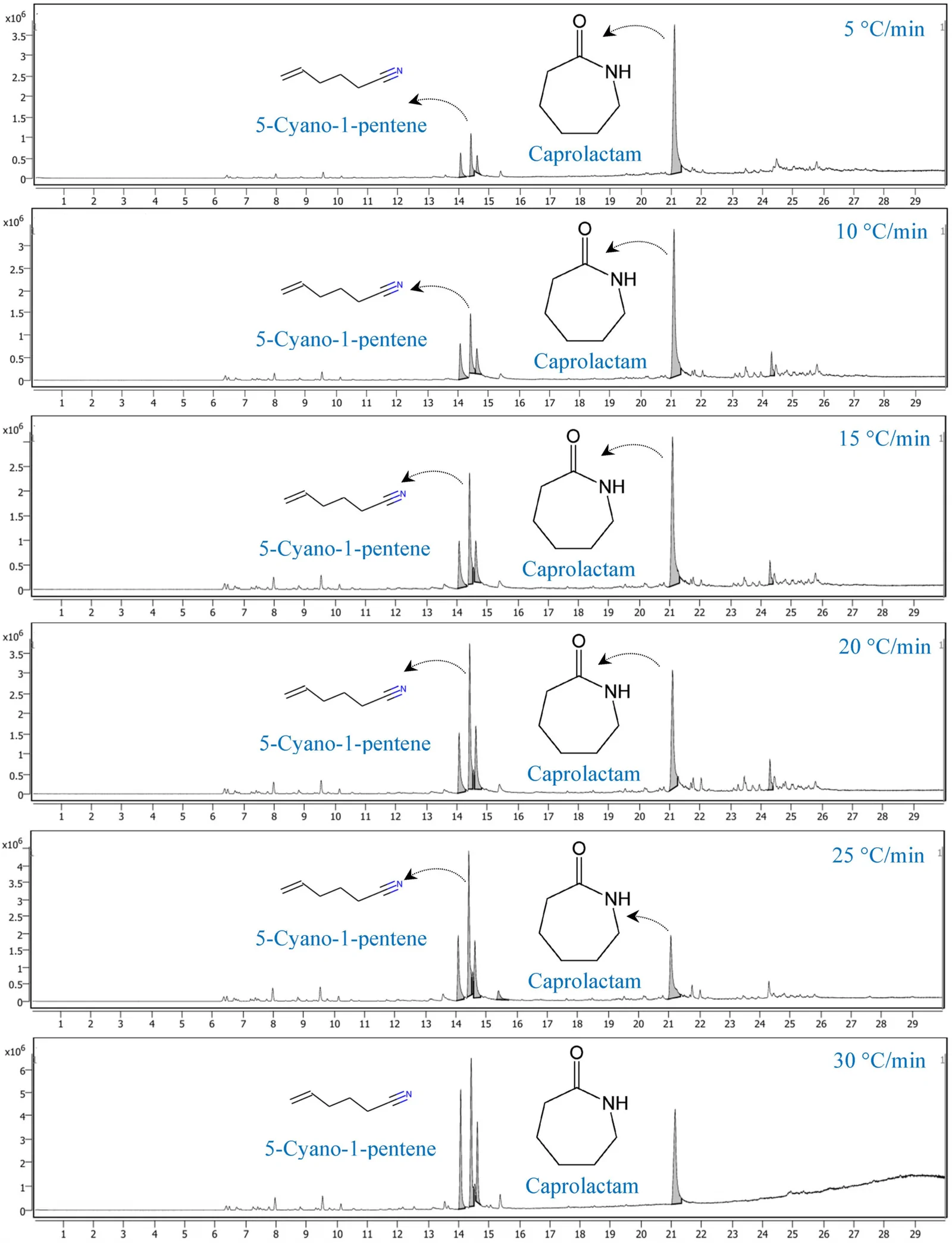
GC/MS chromatogram of the evolved vapors from the catalytic decomposition of the CuFNy sample.

Figure 3 and Figure 4 show the GC/MS spectra of the evolved catalytic pyrolysis vapors from CuFNz and CuFNy samples, and their peak composition and quantity (relative peak areas) are defined in Table 2 and Table 3. The analysis indicated that caprolactam was the dominant chemical compound in the CuFNz sample, and its maximum yield was 87.25 (5 °C/min) and 88.21% (20 °C/min), while 1H-Indole-3-carboxaldehyde 7-methyl- (C_10_H_9_NO) was the major compound in the other GC/MS composition, with its abundance reaching 12.75% (5 °C/min). In the case of the CuFNy sample, the composition of vapors changed a lot, where the maximum caprolactam was less than that obtained from the CuFNz sample in the range of 25.92% (25 °C/min)–75.19% (5 °C/min). It was also noted that 5-Cyano-1-pentene (C_6_H_9_N) was present in significant amounts, ranging from 24.82% at (5 °C/min) to 70.43% at 25 °C/min. These findings confirm that the structure of the catalyst used and the applied heating step play a crucial role in determining the composition of the released catalytic pyrolytic vapors. Since caprolactam is the basic chemical compound of the nylon polymer used in the production of FN as well [35,41,66], these results prove that the catalytic thermochemical treatment can be applied to recover it again as a renewable and cheaper source. Also, the CuFN catalytic pyrolysis has more potential in the extraction of caprolactam (88.21%) compared to the traditional pyrolysis of FN (60%) and CuFN (89.45%), or even the catalytic pyrolysis of FNW in the presence of a ZSM-5 catalyst (83%) [41,46]. In addition, the 5-Cyano-1-pentene compound has huge applications in organic synthesis reactions, in synthesizing organic compounds (ketones, esters, carboxylic acids, etc.), as a solvent, and as a catalyst in several chemical processes [67].

### 3.4. Catalytic Thermal Decomposition Pathway of CuFN

**Figure 5:**
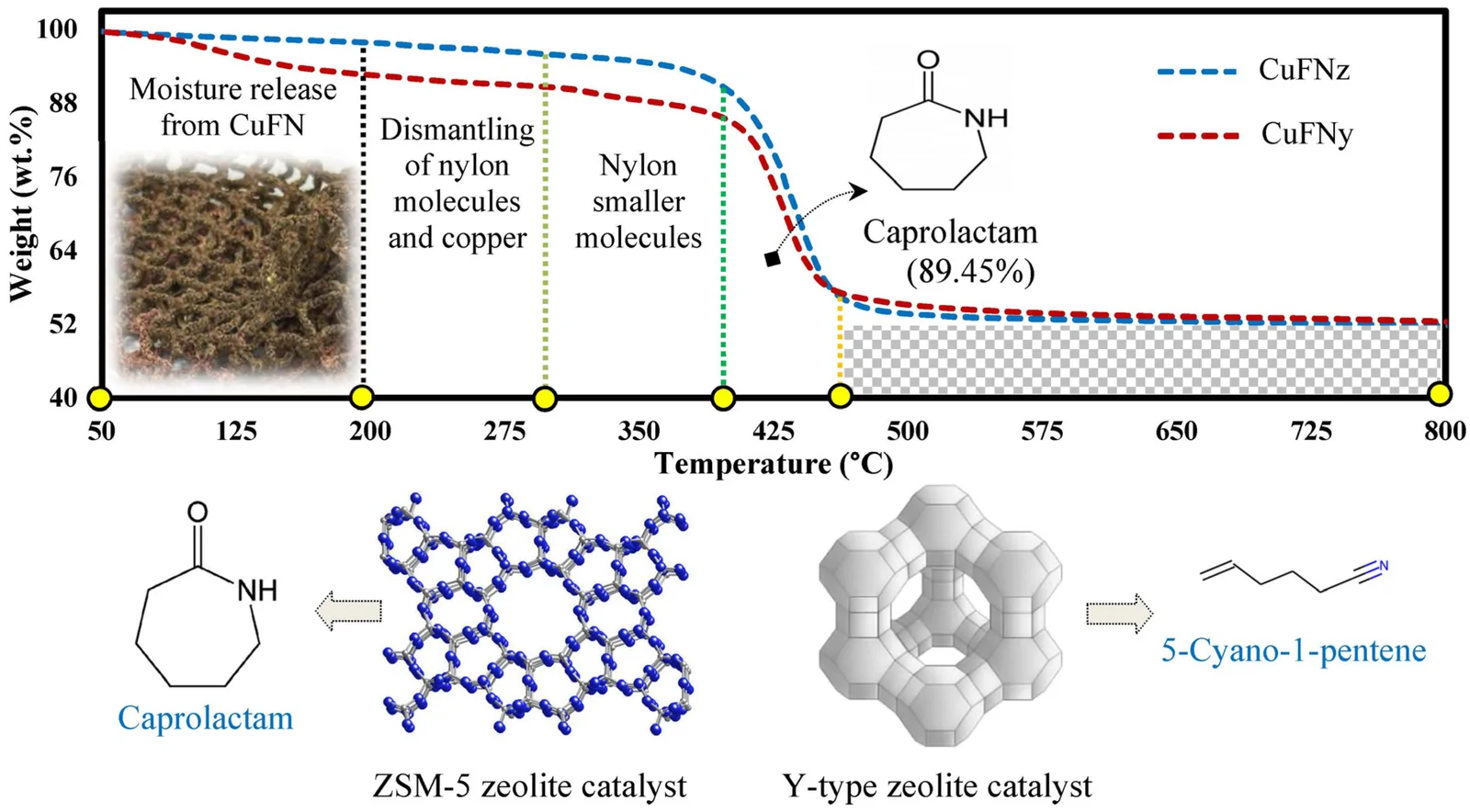
Schematic sketch of the catalytic pyrolysis mechanism of CuFNz and CuFNy.

The nylon polymeric material used in CuFN production is typically manufactured by caprolactam polymerization [66]. The GC/MS results demonstrate that catalytic pyrolysis can be used to extract it with a yield reaching 88.21% (20 °C/min) in the case of CuFNz, while in the CuFNy sample, the caprolactam yield decreased significantly up to 25.92% (25 °C/min)–75.19% (5 °C/min). Also, it was noted that a new compound was synthesized from the decomposition of CuFNy, with a high yield in particular of the 5-Cyano-1-pentene compound, up to 70.43% (25 °C/min). This section was prepared to understand the pathway and catalytic thermal decomposition mechanism of both samples (CuFNz and CuFNy). The catalytic thermal decomposition pathway of CuFN is discussed based on TGA measurements supported by GC/MS measurements, and the results are formulated in Figure 5. The schematic was focused on only TGA profiles of CuFNz at 20 °C/min and CuFNy at 25 °C/min, as they showed the maximum amount of caprolactam (88.21%) and 5-Cyano-1-pentene compound, respectively. As shown, the CuFNz sample exhibited higher thermal resistance up to 200 °C, with small degradation due to moisture release, unlike the CuFNy sample, which showed early degradation starting at around 100 °C and continuing up to 200 °C to release its moisture content. The significant drop at this stage is likely due to the elimination of water that was adsorbed during the reaction [68]. After that, the applied temperature started to spread between CuFN molecules and dismantle nylon molecules and copper debris up to 300 °C [69]. With increasing temperature, its diffusion rate increased too, which allowed it to move from the external circumference of CuFN molecules to the interior, followed by fragmenting nylon particles into smaller molecules until 385 °C [70]. Subsequently, active catalytic pyrolysis was conducted for both CuFN samples. During this stage, the long polymer chains of nylon break down into smaller fragments through random chain scission reactions, promoting depolymerization and converting the feedstock back into its ε-Caprolactam monomer, especially caprolactam [71,72]. The catalysts used help promote the synthesis of the compounds and facilitate the reaction through dehydroxylation and cracking processes. However, it was noted that a Y-type zeolite catalyst provides another composition (5-Cyano-1-pentene) given its enhanced porosity and acidity, which permitted the caprolactam compound to diffuse into its structure, followed by decomposition into 5-Cyano-1-pentene [73,74,75]. Although the separated copper debris does not decompose or participate in the reaction, it can act as a heat transfer substrate that can be used to accelerate heat and pass through the nylon molecules, allowing heat to be retained and reducing the leakage energy (Ea) used in the decomposition process, resulting in improved decomposition, especially when compared to virgin nets [41,46]. After the full breakdown of the nylon polymer material, the solid material remaining in the final stage is called the by-product, and it contains undecomposed copper debris [76].

### 3.5. Catalytic Pyrolytic Activation Energies

**Figure 6:**
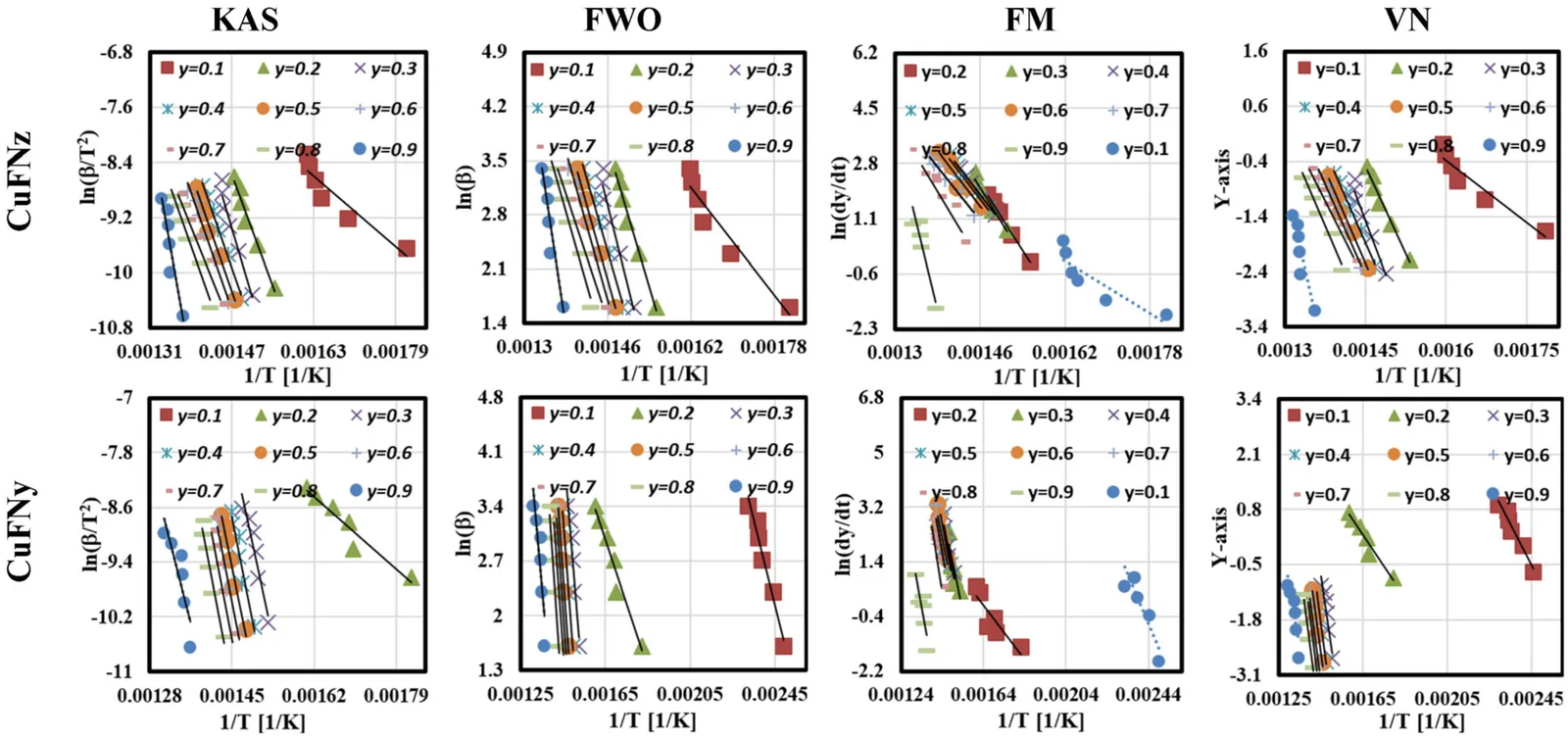
Catalytic pyrolysis kinetics fitting curves of the CuFNz and CuFNy samples.

**Figure 7:**
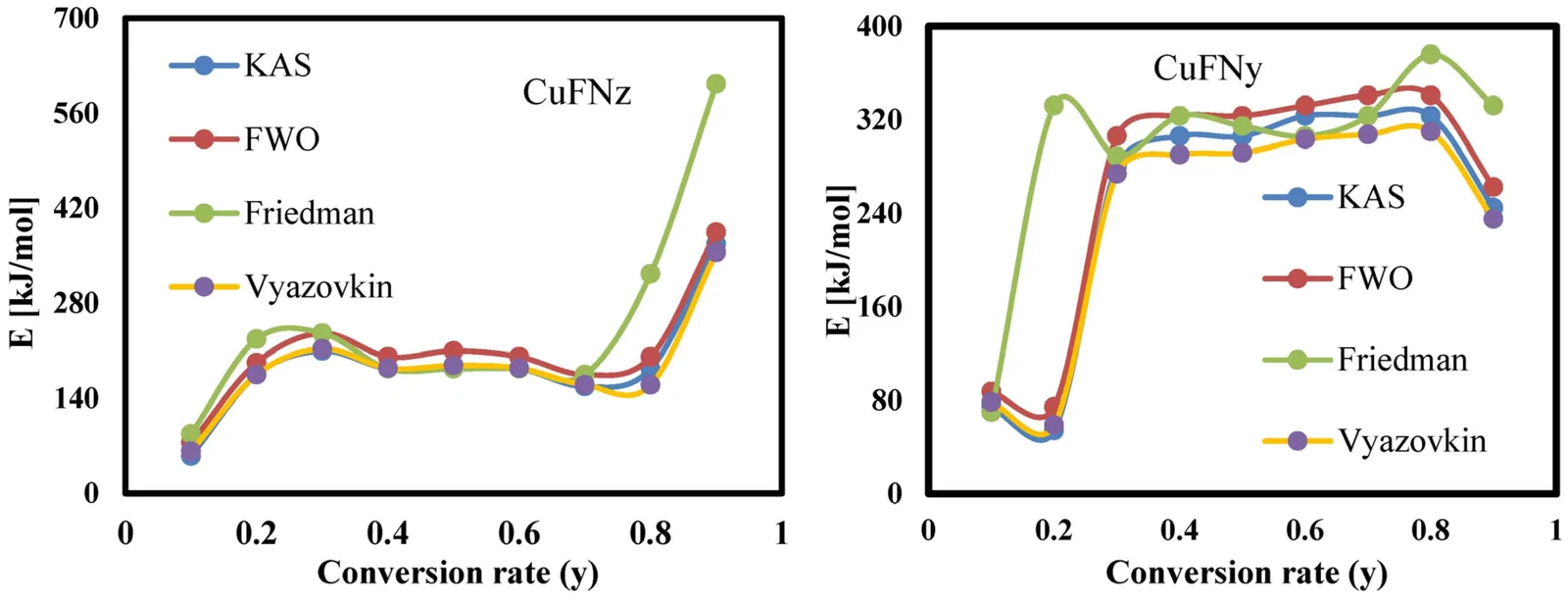
Activation energy distribution of the catalytic pyrolysis of the CuFNz and CuFNy samples.

The activation energies of the catalytic decomposition of CuFNz and CuFNy samples were estimated using model-free techniques using KAS, FWO and FM for curve fitting (Figure 6) with the specified relationships with the help of Equations (2)–(4), and then their slopes were extracted, allowing Ea to be calculated at each conversion rate. As illustrated, the KAS, FWO, and FM plots feature nine lines, corresponding to the decomposition of CuFN samples at conversion rates ranging from 0.1 to 0.9. It is also observed that these lines, in the case of the KAS and FWO models, are very close and parallel in the conversion rate range of 0.2 to 0.8, where the reaction at a low conversion rate is more complex and unstable due to the development of many simultaneous reactions, which makes it difficult to predict them at the same time [77]. Moreover, certain features may also appear at the end of the reaction, along with the formation of a solid carbon fragment, which introduces some variation at the extreme conversion point [78]. Concerning the FM approach, significant randomness was noted in the arrangement of fitted lines due to the presence of fatness, as this approach is highly sensitive to simulated distorted data, which in turn impacts its fitting performance and the reliability of the computed Ea [79,80]. From the calculated slopes, the average values of the Ea item of the CuFNz sample were determined to be 188.8 kJ/mol (KAS: R^2^ = 0.95), 208.4 kJ/mol (FWO: R^2^ = 0.96), and 244.8 kJ/mol (FM: R^2^ = 0.93), while the Ea of the CuFNy sample was 248.4 kJ/mol (KAS: R^2^ = 0.94), 265.7 kJ/mol (FWO: R^2^ = 0.93), and 296.3 kJ/mol (FM: R^2^ = 0.83), as shown in Figure 7 and Table 4. The CuFNy sample consumed more energy for decomposition in the range of 17–24% due to the development of more reactions to transfer caprolactam to the 5-cyano-1-pentene compound, as mentioned above in Section 3.3. Some variation in the calculated Ea values was also observed, with KAS and FWO models providing somewhat similar values, with higher R^2^ values in the range of 0.93–0.96, while the FM model gave much larger values compared to the other models, with lower R^2^ values in the range of 0.83–0.93. This difference is due to the different mathematical mechanism used in modeling each model [81], which leads to this variation. Regarding the VN model, the calculations showed that the estimated average Ea of the CuFNz sample is 186.7 kJ/mol with R^2^ = 0.97 after several iterations, as shown in Table 5. The CuFNy sample was assigned a value of 238.8 kJ/mol, with an R^2^ of 0.92 (Table 3). These values are very close to those obtained using KAS, which means both models are recommended for the simulation of the catalytic pyrolysis of CuFN. However, these results are a little bit higher than those obtained from the typical pyrolysis of CuFN, which falls within the range of 167–193 kJ/mol [46]. This complexity can be explained by the high energy required to separate the copper and the energy required for the upgrading process.

### 3.6. Thermodynamic Analysis

The thermodynamic parameters of CuFNz and CuFNy samples were estimated with Equations (6)–(8), and the corresponding results are listed in Table 6. The results indicated that the thermodynamic parameters for the CuFNz sample fall within the ranges of 242.5–290 kJ mol^−1^ (ΔH), 24.6–158.8 Jmol^−1^K^−1^ (ΔS), and 148.4–224.9 kJ mol^−1^ (ΔG). In the case of the CuFNy sample, these values are 188.8–238.9 kJ mol^−1^ (ΔH), −0.275 to −355 Jmol^−1^K^−1^ (ΔS), and 377.5–487.6 kJ mol^−1^ (ΔG). CuFNy exhibits the lowest ΔH and ΔS values, which means that the reaction is stable, exothermic, and associated with a significant decrease in the system’s disorder and randomness [46,82].

### 3.7. Artificial Neural Network Analysis

**Figure 8:**
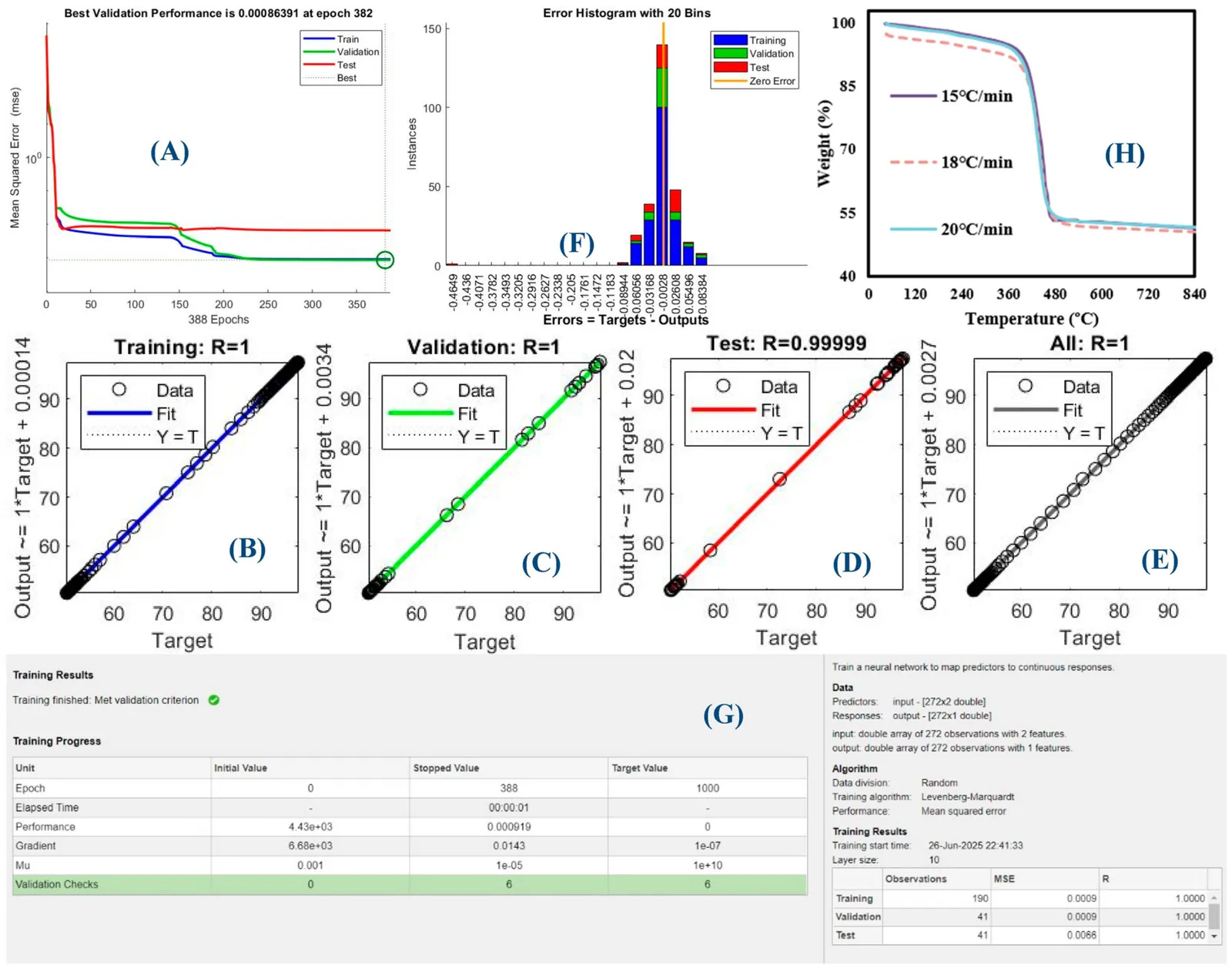
ANN modeling and thermal decomposition characteristics of the CuFNz sample: (**A**) training performance, (**B**–**E**) regression analysis, (**F**) error histogram, (**G**) training results, and (**H**) predicted TGA curves compared with the measured data.

**Figure 9:**
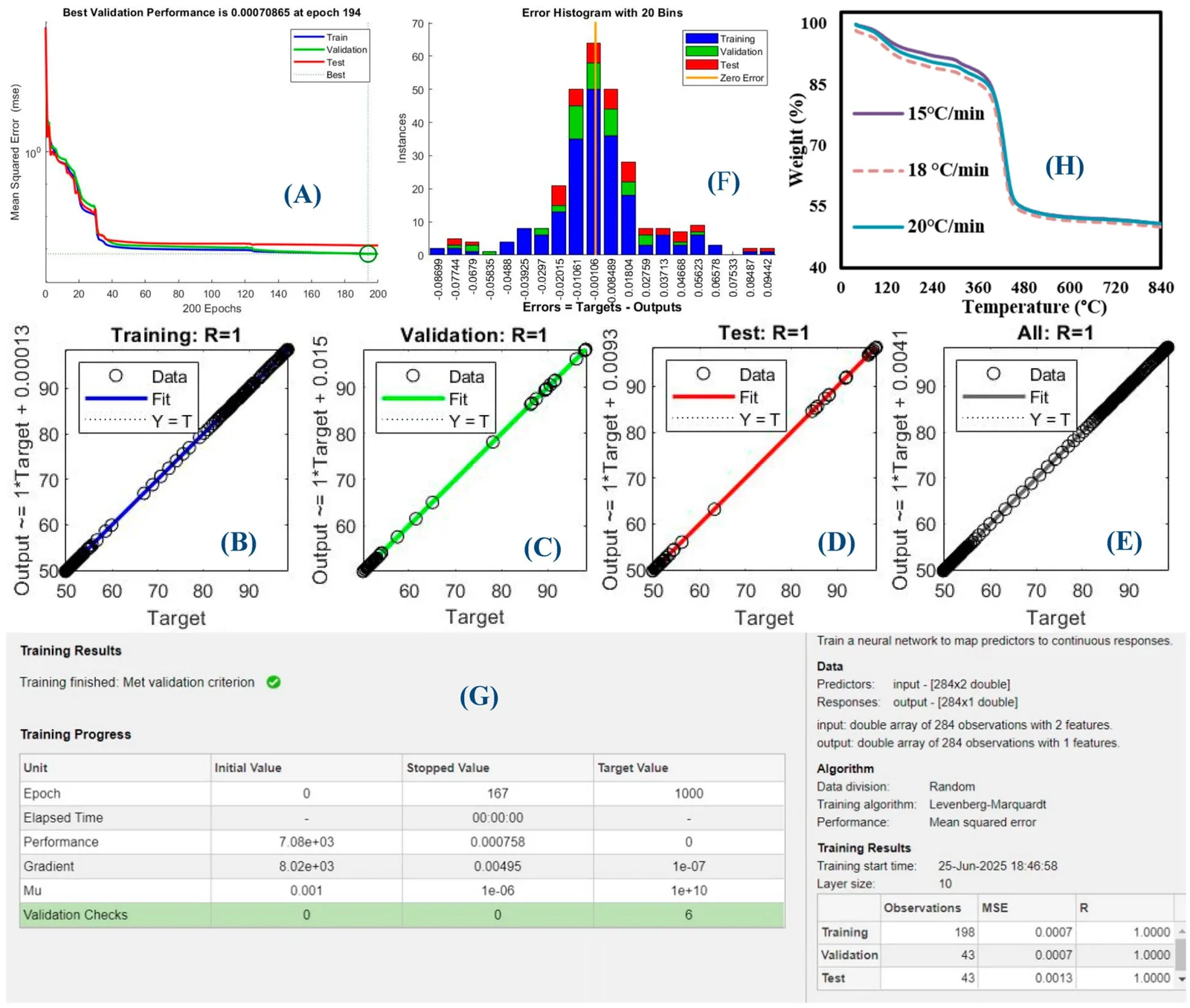
ANN modeling and thermal decomposition characteristics of the CuFNy sample: (**A**) training performance, (**B**–**E**) regression analysis, (**F**) error histogram, (**G**) training results, and (**H**) predicted TGA curves compared with the measured data.

The ANN model, optimized with the LMB configuration and a 3 × 5 × 1 network topology, was employed to predict the CuFN catalytic pyrolysis at a heating rate of 17 °C/min under unspecified conditions. The optimized ANN model demonstrates a swift decrease in MSE, achieving its lowest validation error of 0.00086391 at epoch 382 for the CuFNz sample (Figure 8A) and 0.00070865 at epoch 194 for the CuFNy sample (Figure 9A). The model demonstrates excellent performance, achieving very high R^2^ values (0.99999–1) across the training, validation, and testing datasets for the CuFNz sample (Figure 8B–E) and an R^2^ of 1 for all stages of the CuFNy sample (Figure 9B–E). These results confirm that the LMB configuration is very suitable to optimize the ANN model and record the main degradation regions of catalytic pyrolytic CuFN even when using different catalysts. Meanwhile, the error histogram (at 20 bins) of all stages, including training, validation and testing of both samples, displays a distribution like a normal distribution profile of −0.03168 to 0.02608 (CuFNz (Figure 8F)) and −0.01061 to 0.008489 (CuFNy (Figure 9F)). As shown, the distribution profiles of the two CuFN samples closely follow the zero-error line and remain within the ±1 acceptable error margin for this type of distribution [83], indicating that the ANN model is expected to predict CuFN decomposition with high accuracy. Finally, all training results and their features are summarized in Figure 8G and Figure 9G. Also, these results were used to model the catalytic pyrolysis of CuFNz and CuFNy samples (17 °C/min), and the predictions were fitted against the experimental TGA data for comparison (Figure 8H and Figure 9H). It is clear that the predicted curves match the measured data trend and fall between its limits, which means the ANN model was built well with excellent performance and high simulation capabilities.

## 4. Conclusions

This study investigates the catalytic pyrolysis of copper-incorporated nylon fishing-net (CuFN) waste in the presence of ZSM-5 (CuFNz) and Y-type (CuFNy) zeolite catalysts using thermogravimetric analysis (TGA), thermogravimetric infrared analysis (TG-FTIR), and gas chromatography/mass spectrometry (GC/MS). Kinetic and thermodynamic parameters were extracted from the TGA data, and an optimized artificial neural network model (ANN) was developed to predict the decomposition behavior of both CuFN samples. The TGA showed that CuFNy decomposed at a lower temperature (475 °C) with a higher total weight loss of 52 wt.% compared to CuFNz, which decomposed at 495 °C with a 44 wt.%. Additionally, the CuFNy sample exhibited a narrower and lower maximum decomposition peak temperature range (412–433 °C) relative to CuFNz (409–441 °C). While TG-FTIR analysis of the evolved vapors indicated that the C=O (carbonyl) group was the predominant functional group in the vapors of the CuFNz sample, alkyl C-H stretching was the main functional group in the vapors of the CuFNy sample. Meanwhile, GC-MS analysis showed that caprolactam (88.21% at 20 °C/min) is the main compound of CuFNz’s vapor, while 5-Cyano-1-pentene (70.43% at 25 °C/min) was the major GC/MS compound of vapors that escaped from the CuFNy sample. On the other hand, the CuFNz sample exhibited activation energies between 188.8 and 244.8 kJ/mol (R^2^ = 0.83–0.94), while the CuFNy sample showed higher values, ranging from 248.4 to 296.3 kJ/mol (R^2^ = 0.93–0.97). Finally, the optimized ANN model shows high capabilities in predicting the main decomposition features of both samples, with a high correlation coefficient in training and validation reaching R^2^ = 1. Based on that, catalytic pyrolysis is highly recommended in managing CuFN and decomposing its polymeric part into caprolactam when using a ZSM-5 catalyst and 5-Cyano-1-pentene in the case of a Y-type zeolite catalyst.

## Figures and Tables

**Table 1:** Catalytic thermal decomposition characteristics of CuFNz and CuFNy samples.

Parameter	CuFNz						CuFNy					
Heating rate (°C/min)	5	10	15	20	25	30	5	10	15	20	25	30
$T_{i}$ (°C)	339	341	358	348	334	339	321	344	351	366	357	363
$T_{peak}$ (°C)	409	424	434	441	438	441	412	426	429	432	433	431
$R_{max}$ (%/min)	4.3	7.7	9.4	15.3	17.9	22.6	4.27	7.68	10.37	12.54	17.06	27.23
$R_{avg}$ (%/min)	0.29	0.52	0.67	1.13	1.37	1.62	0.31	0.59	0.86	1.15	1.41	1.81
$M_{f}$ (%)	51.0	55.4	51.5	51.5	53.1	53.6	47.5	49.1	50.5	50.5	51.7	48.1
ΔT_1/2_	34	39	42	41	43	42	34	39	42	41	35	37
CPI (%^3^ °C^−3^ min^−2^)	1.3 × 10^−5^	3.9 × 10^−5^	5.0 × 10^−5^	1.5 × 10^−4^	2.2 × 10^−4^	3.3 × 10^−4^	1.3 × 10^−5^	3.9 × 10^−5^	7.1 × 10^−5^	1.1 × 10^−4^	2.3 × 10^−4^	4.1 × 10^−4^

**Table 2:** GC/MS-identified products from catalytic pyrolysis of the CuFNz sample.

5 °C/min			10 °C/min			15 °C/min			20 °C/min			25 °C/min			30 °C/min		
Time (min)	GC Compound	Area (%)	Time (min)	GC Compound	Area (%)	Time (min)	GC Compound	Area (%)	Time (min)	GC Compound	Area (%)	Time (min)	GC Compound	Area (%)	Time (min)	GC Compound	Area (%)
21.085	Caprolactam	87.25	15.381	4-Pentenenitrile, 2- methylene-	2.60	14.398	Hexanenitrile	7.72	14.415	Hexanenitrile	3.28	14.074	5-Cyano-1-pentene	2.90	14.085	5-Cyano-1-pentene	4.50
24.296	1H-Indole-3 carboxaldehyde, 7- methyl-	12.75	21.115	Caprolactam	85.21	15.367	4-Pentenenitrile, 2- methylene-	7.59	15.378	4-Pentenenitrile, 2- methylene-	3.77	14.411	Hexanenitrile	4.82	14.415	Hexanenitrile	6.58
			24.305	1-Methyl-3-formylindole	12.19	21.126	Caprolactam	77.58	21.102	Caprolactam	88.21	15.377	4-Pentenenitrile, 2- methylene-	3.72	15.381	4-Pentenenitrile, 2- methylene-	6.07
						24.864	5-Imidazol-1-ylmethyl- quinolin-8-ol	7.10	24.320	1-Methyl-3-formylindole	4.74	21.132	Caprolactam	77.37	21.109	Caprolactam	76.30
												24.282	1H-Indole-3- carboxaldehyde, 7- methyl-	7.86	24.286	1H-Indole-3 carboxaldehyde, 7- methyl-	6.55
												25.765	Julolidine	3.33			

**Table 3:** GC/MS-identified products from catalytic pyrolysis of the CuFNy sample.

5 °C/min			10 °C/min			15 °C/min			20 °C/min			25 °C/min			30 °C/min		
Time (min)	GC Compound	Area (%)	Time (min)	GC Compound	Area (%)	Time (min)	GC Compound	Area (%)	Time (min)	GC Compound	Area (%)	Time (min)	GC Compound	Area (%)	Time (min)	GC Compound	Area (%)
14.061	5-Cyano-1-pentene	7.67	14.078	5-Cyano-1-pentene	12.63	14.081	5-Cyano-1-pentene	13.01	14.080	5-Cyano-1-pentene	14.67	14.080	5-Cyano-1-pentene	17.37	14.074	5-Cyano-1-pentene	22.55
14.404	5-Cyano-1-pentene	12.54	14.414	5-Cyano-1-pentene	15.39	14.421	5-Cyano-1-pentene	23.14	14.425	5-Cyano-1-pentene	28.47	14.425	5-Cyano-1-pentene	33.00	14.421	5-Cyano-1-pentene	29.78
14.608	5-Cyano-1-pentene	4.61	14.625	5-Cyano-1-pentene	7.85	14.625	5-Cyano-1-pentene	10.48	14.628	5-Cyano-1-pentene	15.00	14.551	5-Cyano-1-pentene	4.13	14.619	5-Cyano-1-pentene	13.37
21.098	Caprolactam	75.19	21.091	Caprolactam	58.44	21.088	Caprolactam	48.63	21.088	Caprolactam	35.91	14.629	5-Cyano-1-pentene	15.93	21.122	Caprolactam	34.30
			24.289	1-Methyl-3-formylindole	5.68	24.285	1-Methyl-3-formylindole	4.74	24.285	1-Methyl-3-formylindole	5.95	15.401	4-Pentenenitrile, 2-methylene-	3.65			
												21.064	Caprolactam	25.92			

**Table 4:** Catalytic pyrolytic activation energy of the CuFNz and CuFNy samples.

y	*KAS*			*FWO*			*FM*			*VN*	
	*Ea* (kJ/mol)	*A* (1/s)	*R* ^2^	*Ea* (kJ/mol)	*A* (1/s)	*R* ^2^	*Ea* (kJ/mol)	*A* (1/s)	*R* ^2^	*Ea* (kJ/mol)	*R* ^2^
CuFNy sample											
0.1	74.3	5.3 × 10^7^	0.95	87.4	1.4 × 10^14^	0.97	69.9	1.2 × 10^8^	0.82	78.6	0.96
0.2	54.2	1.2 × 10^2^	0.93	74.3	6.0 × 10^8^	0.96	332.2	1.3 × 10^23^	0.78	58.9	0.95
0.3	279.8	9.6 × 10^17^	0.91	306.0	8.5 × 10^24^	0.92	288.5	5.8 × 10^22^	0.77	273.8	0.92
0.4	306.0	1.1 × 10^19^	0.93	323.5	3.6 × 10^25^	0.94	323.5	1.3 × 10^25^	0.88	290.1	0.93
0.5	306.0	2.6 × 10^18^	0.95	323.5	8.4 × 10^24^	0.95	314.7	3.1 × 10^24^	0.89	291.8	0.95
0.6	323.5	1.3 × 10^19^	0.95	332.2	4.3 × 10^25^	0.96	306.0	2.9 × 10^23^	0.86	303.5	0.96
0.7	323.5	9.6 × 10^18^	0.90	341.0	3.2 × 10^25^	0.91	323.5	1.6 × 10^24^	0.82	307.5	0.91
0.8	323.5	7.4 × 10^18^	0.93	341.0	2.4 × 10^25^	0.94	375.9	1.2 × 10^24^	0.88	309.9	0.94
0.9	244.8	6.6 × 10^11^	0.98	262.3	8.7 × 10^20^	0.86	332.2	6.4 × 10^21^	0.76	235.2	0.78
Avg.	248.4	5.0 × 10^18^	0.94	265.7	1.7 × 10^25^	0.93	296.3	2.2 × 10^24^	0.83	238.8	0.92
CuFNz sample											
0.1	55.1	5.5 × 10^2^	0.87	74.3	2.4 × 109	0.93	87.4	8.9 × 10^8^	0.77	61.6	0.97
0.2	174.9	3.3 × 10^10^	0.98	192.3	1.1 × 10^17^	0.98	227.3	5.9 × 10^18^	0.99	174.7	1.00
0.3	209.8	5.9 × 10^12^	0.95	236.1	1.9 × 10^19^	0.96	236.1	7.1 × 10^18^	0.96	212.8	0.97
0.4	183.6	8.2 × 10^9^	0.99	201.1	2.7 × 10^16^	1.00	183.6	1.3 × 10^15^	1.00	184.3	1.00
0.5	183.6	5.3 × 10^9^	0.98	209.8	4.7 × 10^16^	0.98	183.6	8.6 × 10^14^	0.95	188.4	0.99
0.6	183.6	1.3 × 10^9^	0.98	201.1	1.2 × 10^16^	0.98	183.6	6.0 × 10^14^	0.91	184.3	0.98
0.7	157.4	1.8 × 10^7^	0.99	174.9	5.9 × 10^13^	0.99	174.9	5.9 × 10^13^	0.98	159.6	0.99
0.8	183.6	2.8 × 10^8^	0.96	201.1	2.5 × 10^15^	0.97	323.5	3.7 × 10^17^	0.90	159.6	0.96
0.9	367.2	3.2 × 10^20^	0.87	384.7	1.0 × 10^27^	0.89	603.3	8.2 × 10^40^	0.88	355.0	0.89
Avg.	188.8	3.5 × 10^19^	0.95	208.4	1.2 × 10^26^	0.96	244.8	9.1 × 10^39^	0.93	186.7	0.97

**Table 5:** Ea values of the CuFNz and CuFNy samples calculated using the VN model.

y	CuFNy Sample				CuFNz Sample			
	First Iteration	Second Iteration	Third Iteration	Fourth Iteration	First Iteration	Second Iteration	Third Iteration	Fourth Iteration
0.1	78.7	104.4	78.6	78.6	61.7	137.0	61.6	61.6
0.2	59.2	58.9	58.9	58.9	174.7	174.7	174.7	174.7
0.3	273.7	273.8	273.8	273.8	212.8	212.8	212.8	212.8
0.4	290.0	290.1	290.1	290.1	184.3	184.3	184.3	184.3
0.5	291.7	291.8	291.8	291.8	188.4	188.4	188.4	188.4
0.6	304.1	304.2	303.5	303.5	184.3	184.3	184.3	184.3
0.7	308.0	308.1	307.5	307.5	159.7	159.6	159.6	159.6
0.8	310.1	309.9	309.9	309.9	183.6	184.3	159.6	159.6
0.9	234.9	235.0	235.2	235.2	355.2	355.4	355.0	355.0
Average	238.9	241.8	238.8	238.8	189.4	197.9	186.7	186.7

**Table 6:** Thermodynamic parameters of the CuFNz and CuFNy samples.

y	KAS			FWO			FM		
	ΔH (kJ mol^−1^)	ΔG (kJ mol^−1^)	ΔS (Jmol^−1^K^−1^)	ΔH (kJ mol^−1^)	ΔG (kJ mol^−1^)	ΔS (Jmol^−1^K^−1^)	ΔH (kJ mol^−1^)	ΔG (kJ mol^−1^)	ΔS (Jmol^−1^K^−1^)
CuFNz sample									
0.1	68.36	148.77	−112.62	81.46	74.09	10.32	63.96	139.52	−105.82
0.2	48.26	205.83	−220.68	68.36	134.37	−92.44	326.26	196.32	182.00
0.3	273.86	214.06	83.76	300.06	145.30	216.75	282.56	157.41	175.29
0.4	300.06	225.78	104.04	317.56	154.23	228.76	317.56	160.28	220.29
0.5	300.06	234.34	92.04	317.56	162.87	216.66	308.76	159.99	208.37
0.6	317.56	242.29	105.42	326.26	161.88	230.23	300.06	165.35	188.67
0.7	317.56	244.09	102.90	335.06	172.43	227.78	317.56	172.72	202.87
0.8	317.56	245.64	100.74	335.06	174.14	225.38	369.96	226.82	200.48
0.9	238.86	263.29	−34.22	256.36	156.14	140.37	326.26	214.19	156.96
Avg.	242.46	224.90	24.60	259.75	148.38	155.98	290.33	176.96	158.79
CuFNy sample									
0.1	49.23	110.22	−86.39	68.43	148.61	−113.57	81.53	174.81	−132.12
0.2	169.03	349.81	−256.06	186.43	384.59	−280.68	221.43	454.59	−330.25
0.3	203.93	419.60	−305.48	230.23	472.19	−342.71	230.23	472.19	−342.71
0.4	177.73	367.21	−268.38	195.23	402.19	−293.15	177.73	367.20	−268.36
0.5	177.73	367.21	−268.38	203.93	419.59	−305.47	177.73	367.20	−268.36
0.6	177.73	367.21	−268.38	195.23	402.19	−293.15	177.73	367.20	−268.37
0.7	151.53	314.81	−231.28	169.03	349.80	−256.05	169.03	349.80	−256.05
0.8	177.73	367.21	−268.39	195.23	402.19	−293.15	317.63	646.99	−466.51
0.9	361.33	734.38	−528.40	378.83	769.37	−553.17	597.43	1206.54	−862.76
Avg.	188.80	377.52	−275.68	208.40	416.75	−303.45	238.94	489.61	−355.06

## Data Availability

The original contributions presented in this study are included in the article. Further inquiries can be directed to the corresponding author.
